# Outcomes and Complications of Free Anterolateral Thigh Flaps in Lower Limb Reconstruction: A Systematic Review and Comparative Analysis

**DOI:** 10.3390/jcm15145447

**Published:** 2026-07-11

**Authors:** Sarah Reisinger, Karl Heinz Bürger, Klaus Mayr, Andreas Salagean, Carina Anzinger, Christoph Hirnsperger, Georg Mattiassich

**Affiliations:** 1 Medical Faculty, Johannes Kepler University, 4040 Linz, Austria; 2 Department of Trauma Surgery, AUVA Unfallkrankenhaus Linz, 4010 Linz, Austria; heinz.buerger.ordi@gmail.com (K.H.B.); klaus.mayr@auva.at (K.M.); andreas.salagean@auva.at (A.S.); carina.anzinger@auva.at (C.A.); georg.mattiassich@auva.at (G.M.); 3 Department of Trauma Surgery, AUVA Unfallkrankenhaus Salzburg, 5010 Salzburg, Austria; christoph.hirnsperger@auva.at

**Keywords:** anterolateral thigh flap, lower limb reconstruction, surgical outcomes, systematic review

## Abstract

**Background/Objectives**: Reconstruction of complex soft-tissue defects in the lower extremity remains a significant challenge in reconstructive surgery, necessitating reliable vascularized tissue coverage when local options are exhausted. Since 1984, the free ALT flap has been recognized for its versatility, consistent vascular anatomy and low donor-site morbidity. **Methods**: A two-pronged approach was used, consisting of a systematic review of 18 clinical studies (published 2015–2025, *n* > 3400) and a retrospective analysis of 20 patients treated for high-energy traumatic defects at a single center (2017–2021). Primary endpoints included flap survival, complication rates, and functional outcomes. **Results**: The retrospective cohort demonstrated a 90% flap survival rate, which was not significantly different from the figure of 92.4% reported in the literature (*p* = 0.661). The overall complication rate was 40%, significantly higher than the 7.4% literature average (*p* < 0.001). Most complications were localized wound-healing disorders, such as partial necrosis, associated with the zone of injury in trauma. Despite these complications, a 100% limb salvage rate was achieved. Good functional outcomes, defined as regaining weight-bearing capacity, were observed in 80% of patients, consistent with the literature benchmarks (84.8%, *p* = 0.532). **Conclusions**: Within the limitations of this retrospective cohort and systematic review, the findings suggest that the free ALT flap is a reliable option for lower-extremity reconstruction, providing high flap survival and favorable functional outcomes despite a relatively high rate of localized wound complications in complex trauma cases.

## 1. Introduction

Within contemporary microsurgery, free tissue transfer is considered the gold standard for managing extensive tissue loss resulting from high-energy trauma, oncological resection, or persistent infection [1,2,3]. Among available reconstructive options, commonly used free flaps include the ALT, latissimus dorsi, gracilis, and radial forearm flaps, with flap selection depending on defect size, tissue requirements, donor-site morbidity, and patient-specific factors [1,3,4,5,6,7]. Advances in microsurgical reconstruction have greatly improved the treatment of complex soft-tissue defects of the lower extremity over time. Although smaller defects can often be managed with local flaps, larger injuries require free tissue transfer to achieve stable and well-vascularized coverage. As a result, free flap reconstruction has become a cornerstone of modern limb salvage, helping to preserve function and reduce the risk of major complications [1,3,8,9]. The ALT flap has emerged as a primary technique due to its exceptional reliability. Since its initial description by Song et al. in 1984, the ALT flap has become an integral part of reconstructive surgery, valued for its versatility and reliable vascularity and low donor-site morbidity [1,2,3,10].

Reconstruction of lower-extremity defects is particularly challenging due to limited local tissue availability and the need to restore both mechanical function and aesthetic contour. The ALT flap addresses these challenges by providing a long vascular pedicle, enabling microvascular anastomosis outside the immediate zone of injury. This feature is especially important in traumatic cases where recipient vessels near the defect may be compromised.

The clinical utility of the ALT flap is based on its adaptability. It can be harvested in multiple configurations, including musculocutaneous, suprafascial and subfascial dissections, thereby enabling tailored adaptions to the volumetric and structural requirements of the recipient site [4,11,12,13,14]. Additionally, intraoperative thinning or the use of chimeric designs is advantageous for reconstruction of the distal leg and foot, where excessive bulk may hinder footwear and joint mobility [15,16,17]. This study compares outcomes from a single-center retrospective cohort at AUVA UKH Linz with international published reference data to validate the reliability of the ALT flap in complex trauma cases [2,9]. The aim of this study was to systematically review the current evidence on free ALT flap reconstruction for lower-extremity defects and to compare these findings with the outcomes of a retrospective cohort treated at our institution.

## 2. Materials and Methods

### 2.1. PICO Framework

The study structure followed the PICO model to organize the systematic review and comparative analysis [18]:

- Population (P): Patients requiring soft tissue reconstruction of the lower limb [18].
- Intervention (I): Reconstruction using free ALT flaps [18].
- Comparison (C): Reconstruction with alternative free flap techniques (e.g., latissimus dorsi, radial forearm, or gracilis flaps) [18].
- Outcomes (O): Clinical and functional parameters, including flap survival, complication rates, and functional recovery (defined as regaining weight-bearing capacity and resuming daily activities) [18].

### 2.2. Systematic Review Protocol

This systematic review was performed in accordance with the PRISMA 2020 (Table S2) guidelines. No review protocol was registered prior to study initiation. The methodological quality and risk of bias of the included studies were assessed using the appropriate Joanna Briggs Institute Critical Appraisal Checklist according to the respective study design. Based on the overall assessment across domains, studies were categorized as having low, moderate, or high risk of bias. One reviewer independently assessed all included studies. Any disagreements were resolved through discussion until consensus was reached. No automation tools were used during the assessment process.

**Figure 1:**
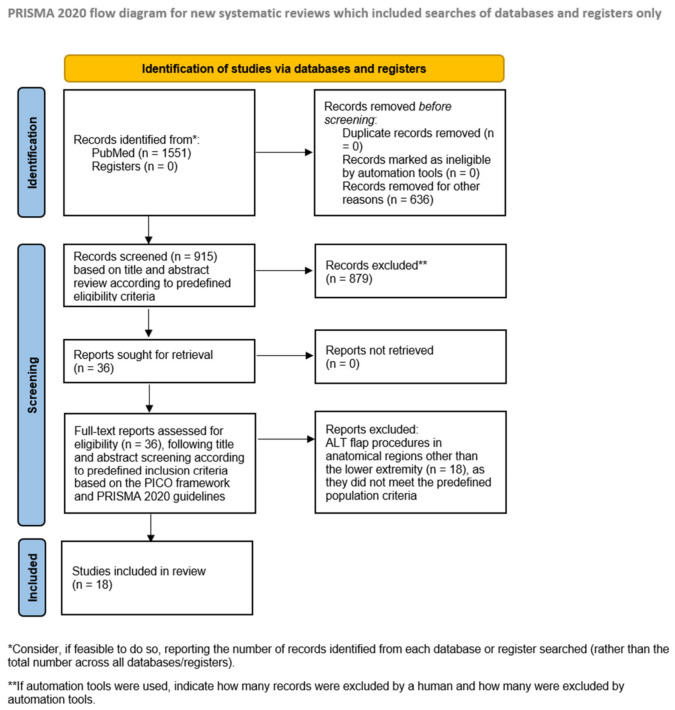
Systematic Literature Search PRISMA Workflow [1,19].

A systematic literature search was conducted exclusively in the PubMed database in January 2026 using the search string “Anterolateral Thigh” AND “flap” AND “lower extremity”. Records were screened by title and abstract, followed by full-text assessment of potentially eligible studies. The search was restricted to the 2015–2025 timeframe. Studies meeting the predefined inclusion criteria were included in the final analysis. Inclusion criteria included clinical trials, meta-analyses, and multicenter studies investigating ALT flaps in the lower body. After filtering for studies specifically addressing the lower extremity, 18 studies representing over 3400 patients were selected for pooled analysis. The included studies were evaluated with regard to the first author, year of publication, study title, study design, sample size, indication for reconstruction, flap type or surgical technique, primary outcomes, and reported complications. Exclusion criteria were studies describing ALT flaps in locations other than the lower body, as well as studies with incomplete or insufficient data (Figure 1).

### 2.3. Retrospective Cohort Analysis

A retrospective review was conducted on 20 patients treated for traumatic soft-tissue defects at the Unfallkrankenhaus (UKH) Linz between 2017 and 2021. The patients included in the retrospective cohort were evaluated for demographic characteristics (age at surgery and sex), indication for reconstruction, defect location, flap type, flap harvest technique, perioperative antibiotic treatment, recipient vessels (artery and vein), previous orthopedic procedures, tobacco use, flap survival, donor- and recipient-site complications, revision surgeries, functional outcomes, and aesthetic results.

Flap elevation was performed using a standardized surgical approach, typically using the medial dissection route to identify perforators arising from the descending branch of the lateral cutaneous femoral artery (LCFA). The intermuscular septum between the rectus femoris and vastus lateralis served as the primary landmark. When dead space was observed, part of the vastus lateralis muscle was included to compensate for the resulting void. Microvascular anastomoses (end-to-end or end-to-side) were performed under a surgical microscope. Medical records were analyzed for primary outcomes, particularly flap survival categorized as complete survival, partial loss, or total loss, as well as secondary outcomes including localized complications, functional recovery, and aesthetic satisfaction.

### 2.4. Statistical Analysis

Statistical evaluation was conducted using Microsoft Excel and JASP version 0.95.4.0. Descriptive statistics were used to summarize patient characteristics and clinical out-comes. Categorical variables were reported as absolute numbers and percentages. Exact binomial hypothesis tests were used to assess whether the observed proportions of flap survival, donor-site morbidity, and functional outcomes in the retrospective cohort differed significantly from the corresponding pooled proportions reported in the systematic review, which served as the reference values. For each comparison, the null hypothesis was that the observed cohort proportion was equal to the pooled literature proportion. Exact 95% confidence intervals, *p*-values, and statistical significance were calculated.

## 3. Results

### 3.1. Systematic Review Summary

Between 2015 and 2025, 18 clinical studies from various countries that performed ALT flap reconstruction in over 3400 patients were included. The parameters assessed included study title, authors, year of publication, journal, place of origin (city and country), study design, DOI, sample size, indication, surgical technique, primary outcomes, complications, and conclusions. The included studies demonstrate the clinical use of the ALT flap across a wide range of reconstructive procedures. Patient demographics, including age and sex, were not consistently reported. The indications for reconstruction were heterogeneous and largely depended on the anatomical region and the underlying pathology. Trauma represented one of the most frequent indications for lower-extremity reconstruction. Additional indications included defects following oncologic resection, chronic ulcers, plantar soft-tissue defects, and salvage procedures after failed reconstructions. Several studies also evaluated mixed patient cohorts, underlining the versatility of the ALT flap in the management of defects with varying etiologies and complexity. Overall, the predominant indication for reconstruction consisted of soft-tissue defects of the lower extremity, particularly involving the foot, ankle, and plantar region. Some studies also focused on scar quality, reducing flap bulk with cryolipolysis, and the use of keystone flaps when primary donor-site closure was not possible. Considerable variation existed in the methodologies used across the studies, particularly regarding preoperative assessment and surgical technique. Five studies primarily investigated preoperative imaging, whereas most focused on surgical outcomes or comparisons of flap techniques. Perforator mapping was a major topic of interest, with handheld Doppler, color duplex ultrasound, CT angiography, and MR angiography being the most commonly used methods. Several studies also evaluated newer imaging technologies, including indocyanine green angiography, photoacoustic tomography, dynamic infrared thermography, and mixed-reality 3D vascular visualization, to improve surgical planning and reduce intraoperative complications. Most studies assessed the conventional ALT flap, although several described modifications designed for specific reconstructive challenges, such as chimeric, sensate, and composite flaps. A large comparative study involving more than 500 patients found that suprafascial flap elevation resulted in lower donor-site morbidity than the subfascial approach, while maintaining comparable flap survival and functional outcomes. Additional studies examined donor-site management, showing that keystone flaps can provide reliable closure without skin grafting when primary closure is not possible. Additionally, it shows that slight wound edge eversion (0.5 cm) leads to improved scar quality and higher patient satisfaction.

When all the studies were combined, they showed a flap survival rate of 92.4%, an overall complication rate of 7.4%, and good functional results in 84.8% of cases. Considerable heterogeneity existed among the studies, particularly regarding preoperative imaging and flap design. These studies found that using preoperative perforator im-aging helped with better planning and fewer complications. Commonly used techniques for perforator mapping included handheld Doppler, color duplex ultrasonography, CT angiography, and MR angiography. Several studies also investigated newer modalities such as indocyanine green angiography, photoacoustic tomography, dynamic infrared thermography, and mixed-reality 3D vascular visualization to improve perforator identification and optimize preoperative planning. In addition to imaging modalities, several studies evaluated different techniques for ALT flap harvest and donor-site management. A large comparative study including more than 500 patients analyzed suprafascial versus subfascial flap elevation. While flap survival and sensory recovery were comparable between both techniques, suprafascial dissection was associated with lower donor-site morbidity, particularly regarding wound-healing complications and functional impairment (Table 1). The characteristics of the studies included in the systematic review are summarized in Supplementary Table S1.

The risk of bias assessment showed that most randomized controlled trials were of moderate methodological quality. This was mainly due to challenges in allocation concealment and blinding. Observational studies generally demonstrated a moderate-to-high risk of bias, largely reflecting the lack of randomization and limited control of confounding variables. Nevertheless, most studies were strengthened by the use of objective outcome measures and appropriate statistical analyses. The included systematic reviews and meta-analyses were also rated as having a moderate risk of bias, mainly due to heterogeneity among included studies and incomplete reporting of publication bias assessments. A detailed summary of the risk of bias assessment for each included study is provided in Supplementary Table S3.

### 3.2. Retrospective Cohort Outcomes

This study included 20 patients who had ALT free flap reconstruction for traumatic lower leg injuries. The cohort consisted predominantly of male patients (18/20, 90%), with a mean age of 42.4 years (range, 16–77 years). All reconstructions were performed following severe traumatic injuries requiring previous orthopedic treatment, most commonly fracture fixation, repeated debridement, necrosectomy and VAC therapy. In 13 patients (65%), reconstruction was achieved using an isolated free ALT flap, whereas seven patients (35%) required combined reconstruction with an additional free flap because of the complexity of the defect. Most flaps were harvested using a subfascial technique (15/20, 75%), while four patients (20%) received myocutaneous flaps and one patient (5%) received a suprafascial flap. Prophylactic antibiotic therapy was administered in all cases. Overall flap survival was 90% (18/20). Two patients experienced flap failure requiring additional reconstructive procedures. Despite these complications, limb preservation was achieved in all patients. Recipient vessels most commonly involved the anterior or posterior tibial artery and their accompanying veins for microvascular anastomosis. Postoperative complications occurred in eight patients (40%). Reported complications included impaired wound healing, flap fistula, hematoma, wound dehiscence, superficial or distal flap necrosis, flap tip necrosis, and temporary sensory or motor deficits. Recipient-site complications were observed in seven patients (35%) and were generally minor. More severe complications, such as hematoma requiring evacuation, wound dehiscence with exposed osteosynthesis, flap fistula, or flap necrosis, were uncommon and were successfully managed with revision surgery. Donor-site morbidity was low and mainly limited to delayed wound healing. About 65% of patients were satisfied with the appearance of their reconstruction. Some patients experienced temporary bulkiness in the flap, which usually improved on its own over time. Several patients required revision procedures during follow-up. These included hematoma evacuation, secondary wound closure, fistula revision, flap revision for necrosis, septic revision, and planned staged closure. One patient underwent multiple revision procedures because of persistent wound complications before successful wound healing was achieved. Functional outcomes were favorable for the majority of patients and were assessed during routine clinical follow-up according to the patient’s ability to perform activities of daily living, ambulation, and return to work where applicable. Seventeen patients (85%) achieved good functional recovery, regaining weight-bearing ability and returning to their daily activities, whereas three patients (15%) experienced persistent functional limitations. One patient reported residual hyposensitivity within the flap despite otherwise satisfactory limb function. Aesthetic outcomes were also generally favorable and were assessed by the treating surgeons during follow-up based on flap contour, color match, and the need for secondary revision procedures. Fifteen patients (75%) were documented as having a good aesthetic result. Five patients initially presented with flap bulkiness. However, this improved spontaneously during follow-up in four patients, while persistent bulkiness at six months was observed in only one case. Smoking status was documented in ten patients, of those nine (45%) were active smokers and 1 (5%) was non-smoker. Smoking status was not given for the remaining ten patients (Table 2).

### 3.3. Comparative Analysis

Postoperative complications occurred in 8 of 20 patients (40%; 95% CI: 19.1–63.9%), which was significantly higher than the 7.4% (8 of 108 patients) reported in the literature (*p* < 0.001). Good functional outcomes were achieved in 16 of 20 patients in the retrospective cohort (80%; 95% CI: 56.3–94.3%), comparable to 84.8% (56 of 66 patients) in the literature (*p* = 0.532). Flap survival with complete defect coverage was achieved in 18 of 20 patients in the retrospective cohort (90%; 95% CI: 68.3–98.8%). This was similar to the reported 92.4% (61 of 66 patients) in the literature (*p* = 0.661), confirming consistently high success rates of ALT flaps, with total flap loss remaining rare and partial necrosis or wound-healing disorders being the most common complications.

Donor-site morbidity was observed in 3 of 20 patients in the retrospective cohort (15%; 95% CI: 3.2–37.9%) versus 4.4% (3 of 67 patients) in the literature, without a statistically significant difference (*p* = 0.055). At the recipient site 7 of 20 patients in the retrospective cohort (35%) showed complications. Most patients achieved stable soft-tissue coverage and satisfactory recovery, including the ability to bear weight and resume daily activities. Inferior outcomes were mainly associated with injury severity rather than the reconstructive technique.

Aesthetic results were largely consistent with the literature, with good contour and appearance in most cases. Initial flap bulkiness was common but typically improved over time without requiring secondary debulking, reflecting the tendency of fasciocutaneous ALT flaps to undergo spontaneous thinning during follow-up (Table 3).

### 3.4. Representative Case with Favorable Outcome

**Figure 2:**
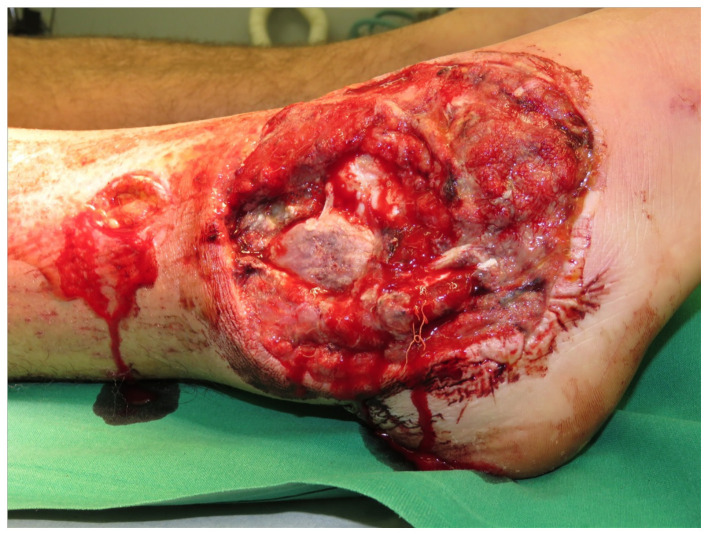
Defect of the right ankle region.

The first case involves a male patient who sustained an open fracture of the right ankle following a traumatic incident (Figure 2). The fracture was initially stabilized using an external fixator, and VAC therapy was applied to the defect. As adequate wound healing could not be achieved with VAC therapy alone, reconstruction was performed using an ALT flap harvested from the right upper leg.

**Figure 3:**
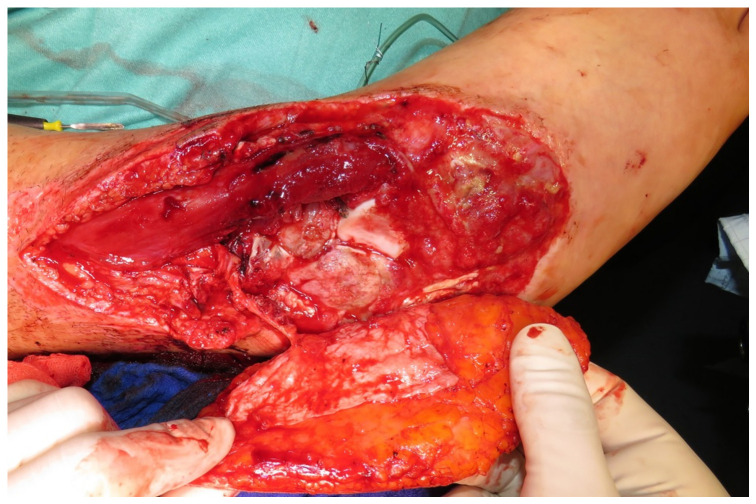
Free subfascial flap prior to inset.

**Figure 4:**
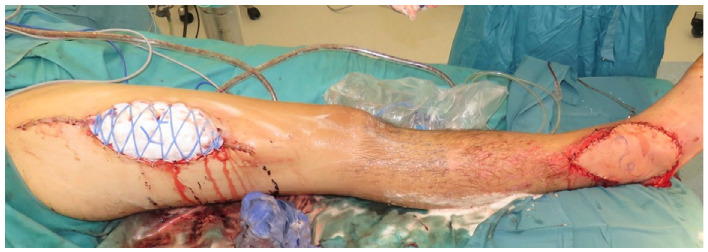
Postoperative result.

**Figure 5:**
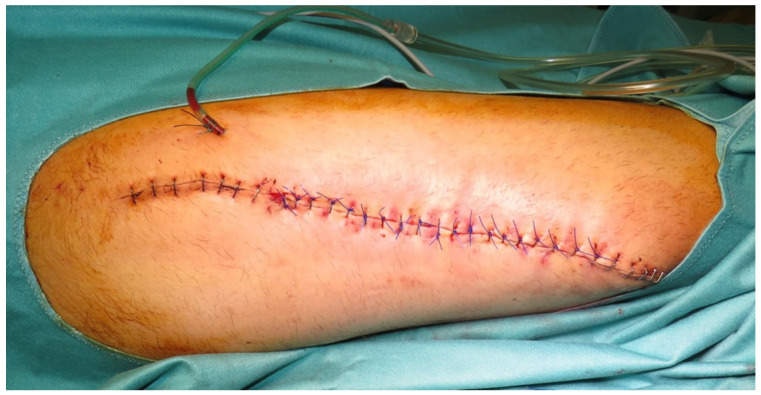
Donor site closure after one week.

**Figure 6:**
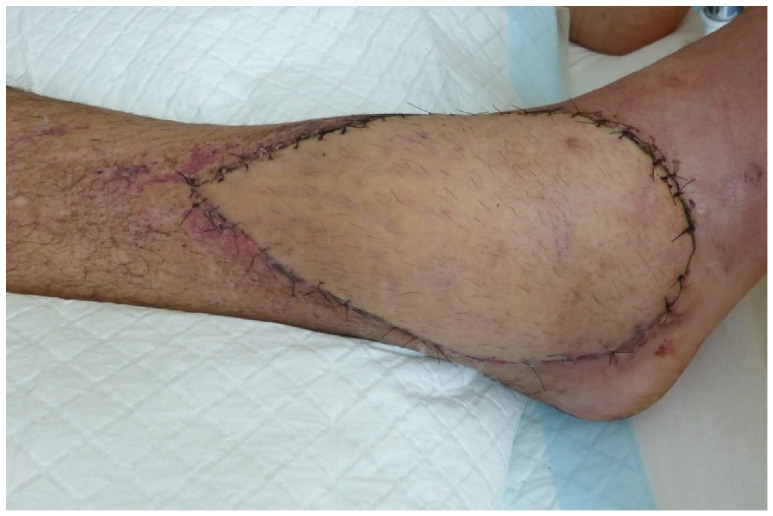
Wound healing after two weeks.

**Figure 7:**
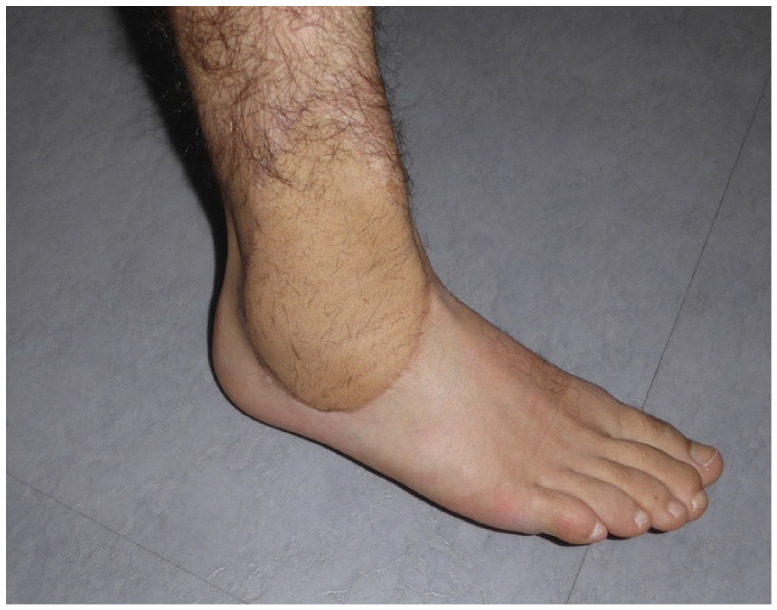
Surgical outcome at one-year follow-up.

A free subfascial flap was microsurgically anastomosed to the anterior tibial vein and an accompanying vein, while arterial inflow was established by connecting the descending branch of the LCFA to the anterior tibial artery. No complications were observed at either the donor or recipient site. The figures below illustrate the course of treatment from the initial surgery to the one-year follow-up (Figure 3, Figure 4, Figure 5, Figure 6 and Figure 7).

### 3.5. Clinical Case with Flap Failure

**Figure 8:**
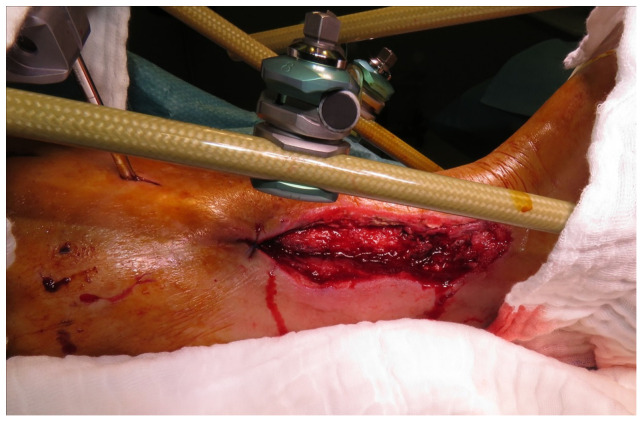
Soft tissue defect after initial trauma.

**Figure 9:**
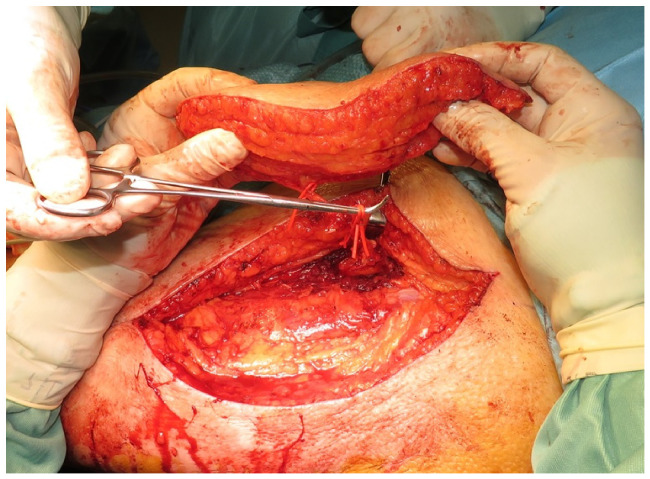
Flap harvest from the donor site.

**Figure 10:**
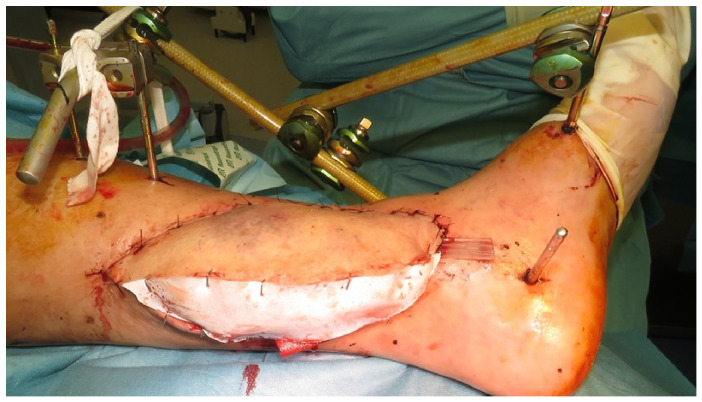
Result after first surgery.

**Figure 11:**
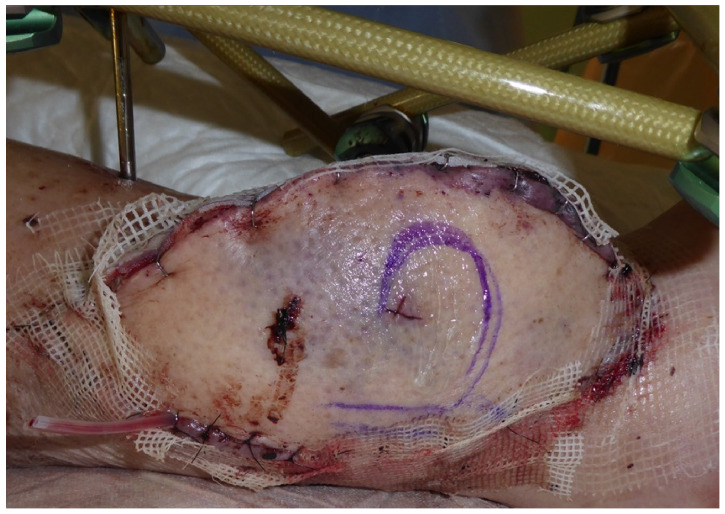
Wound healing after two days.

**Figure 12:**
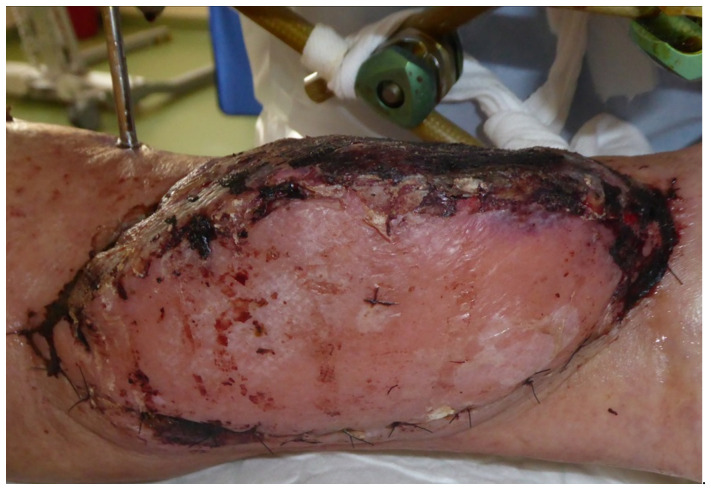
Flap necrosis.

In this case, a female patient was initially treated following trauma resulting in fractures of both lower legs (Figure 8). A VAC device was applied to a soft tissue defect on the left lower leg. However, wound closure could not be achieved. Consequently, reconstruction was performed using a subfascial ALT flap harvested from the left upper leg (Figure 9). The two venous outflows of the flap were anastomosed to the posterior tibial vein and an accompanying vein. Arterial inflow was established via a flap perforator, which was anastomosed to the posterior tibial artery. Two days postoperatively, partial necrosis developed at the distal tip of the flap, necessitating revision surgery with debridement (Figure 10, Figure 11 and Figure 12). The resulting residual defect was subsequently covered with an additional flap.

**Figure 13:**
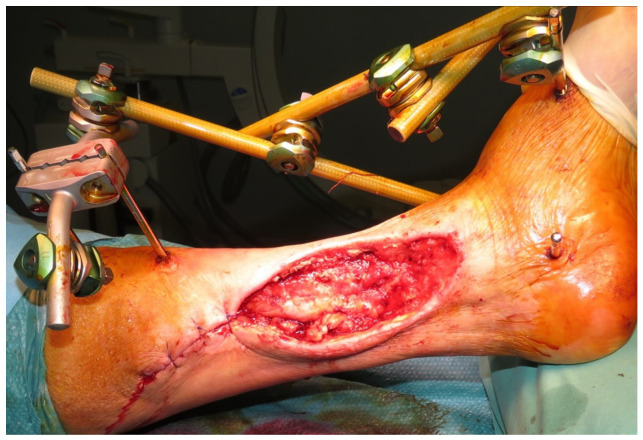
Flap removal after three weeks.

**Figure 14:**
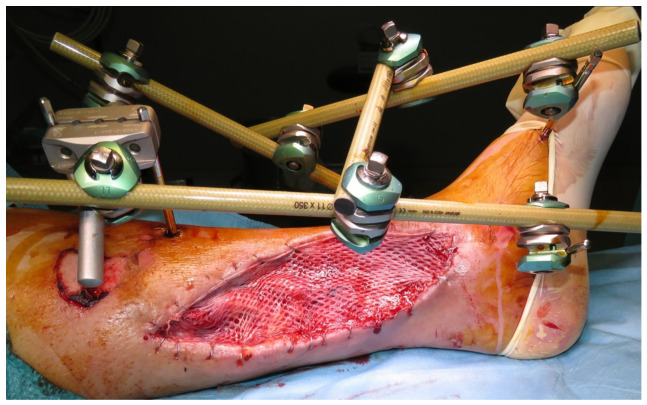
Defect coverage with skin graft.

Despite these measures, progressive flap necrosis occurred, and the flap had to be completely removed three weeks after the initial procedure due to failed healing (Figure 13). The defect was then managed with a split-thickness skin graft in combination with VAC therapy (Figure 14). No complications were observed at the donor site. The patient required a prolonged hospital stay of five months due to wound-healing complications and multiple revision surgeries and additionally developed pressure ulcers at several sites. Functional and aesthetic outcomes were limited, as the initial flap ultimately failed.

## 4. Discussion

This study uses both a systematic literature review and a retrospective clinical analysis to assess the effectiveness of ALT flap reconstruction for complex lower limb defects. The main finding is that patient outcomes in our review closely align with published evidence, demonstrating that the ALT flap consistently delivers reliable results, even in difficult, high-energy injury cases.

The retrospective group consisted exclusively of traumatic cases, where patients often presented with a history of extensive orthopedic and reconstructive surgeries, including external fixation, VAC therapy, necrosectomy and vascular repairs. This represents a particularly challenging patient population, reflecting global trends in which trauma remains a leading indication for free ALT flap reconstruction. The consistency of outcomes between this cohort and the published data suggests that the ALT flap performs reliably even in challenging clinical cases. The 90% survival rate observed in the retrospective cohort did not differ significantly from the figure of 92.4% reported in the international literature (*p* = 0.661). This shows that the ALT flap is a reliable option for microvascular transfer in traumatic skin defects in high volume trauma centers.

In contrast, the overall complication rate was significantly higher in the retrospective cohort compared to published data (40% vs. 7.4%; *p* < 0.001). This divergence may primarily be explained by the patient population treated at UKH Linz, which mainly consists of high-energy trauma cases, whereas the published studies included indications like defects following oncologic resection, trauma or chronic wound-healing disorders. The underlying indication is an important factor when interpreting outcomes. In oncological reconstruction, the primary objective is to restore soft-tissue coverage after radical tumor resection while preserving limb function, whereas traumatic reconstruction is frequently complicated by extensive zones of injury, contamination, and compromised local vascularity. Recent orthoplastic concepts emphasize that successful limb salvage after tumor resection depends on close collaboration between orthopedic oncologists and reconstructive microsurgeons, enabling adequate oncologic resection while facilitating functional soft-tissue reconstruction [20]. In such cases, the zone of injury often extends beyond the visible defect, involving damaged microvascular tissue and compromised surrounding soft tissue. This leads to more localized wound-healing problems, like partial necrosis or marginal dehiscence. However, it is important to separate these manageable localized events from total flap failure. The 100% limb salvage rate in our cohort demonstrates that these complications do not prevent a successful reconstructive outcome.

Functional outcomes were favorable, with 80% of patients achieving good recovery and returning to daily activities, which is consistent with international data (*p* = 0.532). Less favorable outcomes were particularly seen in cases with severe initial trauma, bilateral injuries, or serious consequences following the trauma. In terms of aesthetics, initial flap bulkiness was observed in several cases. However, ALT flaps often adjust their bulky appearance over time. This process reduces the long-term need for secondary surgical debulking, which improves lower leg movement.

The ALT flap is highly adaptable, with options such as thinned or chimeric flaps, making it a strong choice for reconstructing the lower leg and foot. Its long blood vessel supply allows surgeons to connect it to healthy vessels, usually the anterior or posterior tibial arteries. Comparing our data with published studies shows that the ALT flap gives reliable results for different defect sizes and injury levels. Even patients with risk factors like smoking or previous infections had good soft-tissue coverage and overall outcomes. This supports using the ALT flap as a primary option for lower-limb reconstruction after trauma. Although donor-site complication rates were comparable to those reported in previous studies, the slightly higher incidence observed in our cohort may be attributable to the limited sample size and the severity of the injuries. Since all patients were polytrauma cases, this likely contributed to the impaired wound healing.

One of the most clinically relevant aspects of this study is its contribution to the ongoing debate between limb salvage and primary amputation. Traditionally, decision-making has relied on injury severity scores. However, these tools have shown limited predictive value in the era of advanced microsurgical reconstruction. The findings presented here support a shift toward a reconstruction-oriented perspective. While these findings suggest that limb salvage can be feasible even in challenging cases, they should be interpreted in light of the retrospective study design, limited sample size, and the highly selected patient population treated at a specialized microsurgical center.

The data from this study show that complications should not shift the decision toward amputation. Most complications were manageable and did not compromise final limb preservation, emphasizing that limb salvage is often a staged process rather than a single definitive intervention. This concept is consistent with contemporary orthoplastic approaches in musculoskeletal oncology, where microsurgical free tissue transfer has substantially expanded the indications for limb-sparing surgery following tumor resection. In these patients, flap reconstruction not only provides durable soft-tissue coverage but also enables adequate oncological resection without compromising reconstructive options [20]. The versatility, long vascular pedicle, and reliable vascularity of the ALT flap may have contributed to the favorable reconstructive outcomes observed in this cohort. Although the ALT flap is one of the most frequently used free flaps in both traumatic and oncological lower-extremity reconstruction, flap selection should remain individualized according to defect size, tissue requirements, recipient vessels, and functional demands [20]. While the present results are encouraging, they should be interpreted with caution. Successful limb salvage depends on multiple factors, including patient characteristics, injury pattern, timing of reconstruction, and institutional microsurgical expertise, rather than on flap choice alone.

This study is limited by the small size of the retrospective cohort (*n* = 20) and the variety of study designs in the literature review. Furthermore, the systematic review included studies with heterogeneous patient populations, indications, study designs, outcome measures, and follow-up periods, which limits the direct comparability of the published data. In addition, functional outcomes were assessed during regular clinical follow-up visits over a postoperative period of one year rather than using standardized functional scoring systems. These factors should be considered when interpreting the results. Nevertheless, the outcomes observed in our patient cohort were largely consistent with those reported in the literature. By combining our institutional experience with a systematic review of recent evidence, this study provides a comprehensive overview of the clinical performance of the free ALT flap in lower-extremity reconstruction. The findings may help surgeons when selecting an appropriate reconstructive option by offering additional evidence on flap survival, complication rates, donor-site morbidity, and functional outcomes.

## 5. Conclusions

The findings of this study suggest that the free ALT flap is a reliable option for the reconstruction of complex lower-extremity defects. Although localized complications were more frequent in our high-energy trauma cohort than in the published literature, flap survival and functional outcomes were comparable. These findings should be interpreted in light of the retrospective design, small cohort size, and heterogeneity of the included studies. Future research should utilize larger multicenter cohorts and standardized assessment tools to further refine reconstructive protocols.

## Figures and Tables

**Table 1:** Study Characteristics and Outcomes.

Study Number	Study Design	Number of Patients	Indications	Outcomes
1	Clinical trial	25	Plantar Soft Tissue Defect	Good flap contour and stability, full ambulation in all patients at 12 months, partial sensory recovery in reinnervated flaps
2	Systematic review and meta-analysis	672 (CDU group) and 531 (CTA group)	Preoperative imaging for ALT flap planning in reconstructive surgery	CDU had higher perforator detection sensitivity (95.3%) than CTA (90.4%); both had low false-positive rates (CDU: 2.8%, CTA: 2.4%); accuracy in course identification was high in both methods
3	Multicenter study	41	Soft tissue defect location was mainly on the foot and ankle (61%) and bone exposure was found in 82.9% of cases. The indication was trauma in 58.5% of cases	Flap success rate: 92.8%, Functional results rated “very good” or “good” in 75.5% of cases. Aesthetic results rated “very good” or “good” in 63.6% of cases.
4	Clinical trial	8	Preoperative imaging for ALT flap planning in reconstructive surgery	Correlation between PAT-predicted and intraoperative perforator locations, classification of perforator branching patterns
5	Multicenter study	1079	Salvage options for ALT flap reconstruction	28 ALT flaps discarded due to nonviable skin paddle or lack of perforators. Salvage options successfully utilized, with minimal additional morbidity
6	Randomized controlled trial	12	Preoperative perforator mapping for ALT perforator flap	ICGA detected 100% of perforators with highest accuracy—hhD sensitivity: 67%, PPV: 62%; CDU sensitivity: 73%, PPV: 64%
7	Randomized controlled trial	32	Preoperative imaging for ALT perforator flap planning	Thermography reduced ultrasound exam time by 90–130 s, positive correlation between perforator quantity and exam time
8	Meta-analysis	197	Lower limb reconstruction after trauma (42%), oncology (31%), complex wounds (20%), and infection (7%)	Physiological benefit from dangling demonstrated by improved tissue oxygen saturation; early dangling (as soon as POD 3) appears feasible in some cases
9	Meta-analysis	416	Soft-tissue defects requiring free flap soft-tissue reconstruction; the analysis compares thin ALT flaps with other free soft flaps	No significant difference in flap failure and in the need for secondary salvage surgery. ALT flaps exhibited significant advantages compared to other flaps in terms of skin grafting requirement, donor-site morbidity, recipient-site morbidity, and patient satisfaction
10	Systematic review and meta-analysis	Not given	Lower limb reconstruction following trauma	No significant difference in total flap failure, reoperation, or limb salvage rates between muscle and fasciocutaneous flaps. Partial flap failure was lower in fasciocutaneous flaps
11	Randomized controlled trial	80	Preoperative perforator vessel localization for ALT perforator flap surgery	Identification rate of perforators: MR: 94.3%; CDU: 82.0% Accuracy of perforator location: MR: 1.5 mm; CDU: 2.7 mm Secondary outcomes: Flap harvest time: MR: 52 min; CDU: 68 min
12	Clinical trial	7 patients	Soft tissue defects in the head and neck region, and lower limb (defects ranging from 4.0 cm^3^ to 40.0 cm^3^)	Successful reconstruction of soft tissue defects, adjustable flap volume, rich vascular supply, combination with bone and cartilage tissue
13	Clinical trial	36	Lower-extremity reconstruction due to acute/chronic wounds and oncologic resection	23 patients ambulated, 3 began leg dangling on POD1; one flap showed reversible early mottling; all flaps healed successfully
14	Systematic review and meta-analysis	284	Head and neck (53%), lower limb (7.9%) reconstruction after cancer resection and trauma	Flap failure rate: 3.8%; overall complication rate: 38%; emergency return to theatre rate: 19%; flap salvage success: 52%
15	Clinical trial	22	Bulky free adipocutaneous ALT flaps after distal extremity reconstruction	Cryolipolysis reduced extremity circumference (mean −1.8 cm) and fat thickness (−7.7 mm) significantly, 90% patient satisfaction, Shorter hospital stay vs. surgery
16	Meta-analysis	525	Reconstruction of defects	No difference in need for skin grafting or sensory recovery, SPF group had lower rates of poor healing and donor site dysfunction
17	Case report	6	Soft tissue reconstruction using ALT flap in cases where primary closure of the donor site was not possible	Successful donor site closure without skin grafting; preserved hip and knee range of motion
18	Randomized controlled trial	36	Donor site wound closure after ALT flap harvest, focus on scar formation	6 months post-op: Patient and Observer Scar Assessment Scale: Best outcomes in Group B, followed by Group A, worst in Group C, Patient satisfaction (VAS): Highest in Group B, Vancouver Scar Scale (VSS) & scar width: No significant differences among groups

**Table 2:** Patient Characteristics and Surgical Outcomes.

Patient Number	Indication	Summary Characteristics	Flap Survival	Donor Site Complications	Recipient Site Complications	Functional Outcome	Aesthetic Result
1	trauma	Open medial malleolar fracture, operative fixation, VAC therapy	yes	none	none	good	good
2	trauma	Open ankle fracture, external fixation, plating, VAC therapy	yes	none	Flap fistula	Hyposensitivity in flap area	good
3	trauma	Extensive soft-tissue defect, VAC therapy	yes	Delayed donor-site healing	none	good	good
4	trauma	Open lower leg fracture	yes	none	none	good	Initially bulky, improved over time
5	trauma	Polytrauma, external fixation, pelvic fixation, VAC therapy	yes	none	Wound dehiscence requiring debridement and VAC therapy	good	good
6	trauma	Operative fracture fixation	no	none	Partial distal flap necrosis (8 × 2 cm), debridement, VAC therapy (8 days)	good	good
7	trauma	Polytrauma, right upper- and lower-extremity amputation, compartment syndrome requiring fasciotomy, open calcaneal fracture, VAC therapy	yes	none	none	good	good
8	trauma	Tibial intramedullary nailing, fasciotomy, Epigard coverage of the left ankle	yes	Delayed donor-site healing	Flap wound-healing disorder, Epigard applied and removed (4 days)	good	good
9	trauma	Fracture fixation, compartment release, VAC therapy, necrosectomy, toe amputation	yes	none	none	good	Initially bulky, improvement after 4 months
10	trauma	External ankle fracture fixation, VAC therapy	yes	none	none	good	good
11	trauma	External fracture fixation, Epigard coverage, VAC therapy, necrosectomy	yes	none	Superficial flap necrosis	good	Flap bulkiness, reduced after 5 months
12	trauma	Fracture fixation (bilateral lower legs), left lower leg necrosis, VAC therapy	no	none	Flap tip necrosis, secondary flap coverage, complete flap removal	restricted	restricted
13	trauma	Open fracture fixation (K-wire), VAC therapy, necrosectomy	yes	none	none	restricted	Initially bulky, improved over time
14	trauma	External ankle fixation, Epigard coverage	yes	Sensory and motoric lesion (femoral nerve)	Postoperative tense flap with hematoma, revision after 1 day, VAC therapy and small necrosectomy, delayed bone healing treated with shockwave therapy	restricted	good
15	trauma	External lower leg fixation, debridement, Epigard coverage, necrosectomy, subtotal lower leg amputation, VAC therapy	yes	none	none	good	good
16	trauma	Ankle fracture fixation, VAC therapy	yes	none	none	good	Initially bulky, improved over time
17	trauma	Ankle fracture fixation, VAC therapy, vascular repair (A. tib. ant.), necrosectomy + fasciotomy	yes	none	none	good	good
18	trauma	Calcaneus fracture fixation, Achilles tendon repair, necrosectomy, VAC therapy	yes	none	none	good	Persistent bulkiness after 6 months
19	trauma	Necrosectomy, VAC therapy	yes	none	none	good	good
20	trauma	External fracture fixation	yes	none	none	good	good

**Table 3:** Comparison of Cohort Outcomes vs. Systematic Review Data.

Outcome Measure	Retrospective Cohort (*n* = 20)	Literature Data (*n* > 3400)	*p*-Value
	90% (*n* = 18)	92.4% (*n* = 61)	0.661
Overall Complications	40% (*n* = 8)	7.4% (*n* = 8)	<0.001
Good Functional Outcomes	80% (*n* = 16)	84.8% (*n* = 56)	0.532

## Data Availability

The original contributions presented in this study are included in the article. Further inquiries can be directed to the corresponding author.

## Supplementary Materials

The following supporting information can be downloaded at: https://www.mdpi.com/article/10.3390/jcm15145447/s1, Table S1: Characteristics of all studies included in the systematic review; Table S2: PRISMA checklist 2020; Table S3: Risk of bias assessment.

## Abbreviations

The following abbreviations are used in this manuscript:

ALT	Anterolateral Thigh Flap
LCFA	Lateral Circumflex Femoral Artery
VAC	Vacuum Assisted Device
