# Supplementary material for: Outcomes and Complications of Free Anterolateral Thigh Flaps in Lower Limb Reconstruction: A Systematic Review and Comparative Analysis
Source: J Clin Med. 2026 Jul 11;15(14):5447. doi: 10.3390/jcm15145447 (PMC13411515; doi:10.3390/jcm15145447)
Supplement: Supplementary file 1 [file jcm-15-05447-s001.zip › jcm-4389713-supplementary.pdf]

Supplementary Table S1: Characteristics of all studies included in the systematic review:

Study Number	PMID	First Author	Year	Title	Study Type	Sample Size	Indication	Flap Type / Technique	Main Outcomes	Complications	Follow-up Duration	Conclusion	DOI
1	26367370	Olivan	2015	Chimerical anterolateral thigh flap for plantar reconstruction	Clinical Trial	25	Plantar Soft Tissue Defect	chimerical ALT, composed of vastus lateralis muscle segment and thinned skin paddle, neurorrhaphy between lateral femoral cutaneous nerve and calcaneal nerve in 7 patients	Good flap contour and stability; full ambulation in all patients at 12 months; partial sensory recovery in reinnervated flaps	2 patients with partial loss of the skin paddle; no major complications in the remaining cases	6 and 12 months	The chimeric ALT flap is suitable for plantar defect reconstruction, providing good contour, stability, ambulation, and a low complication rate	10.1002/micr.22492
2	28583750	McGhee	2017	Systematic review: Early versus late dangling after free flap reconstruction of the lower limb	Systematic review and meta-analysis	197 patients across 8 studies (1 randomized trial, 6 cohort studies, 1 case series)	Lower limb reconstruction after trauma (42%), oncology (31%), complex wounds (20%), and infection (7%)	Mostly latissimus dorsi; others included parascapular (18%) and anterolateral thigh (15%); remainder mixed	Physiological benefit from dangling demonstrated by improved tissue oxygen saturation; early dangling (as soon as POD 3) appears feasible in some cases	4 flap failures (2.0%) reported across all studies	Not consistently reported across studies in the review	Post-operative dangling has physiological benefits; a 3-day dangling protocol may be sufficient; however, optimal timing and regimen remain uncertain and require further study	10.1016/j.bjps.2017.04.001
3	34959247	Moore	2022	Color Doppler Ultrasound versus Computed Tomography Angiography for Preoperative Anterolateral Thigh Flap Perforator Imaging: A Systematic Review and Meta-Analysis	Systematic review and meta-analysis	672 patients (CDU group) and 531 patients (CTA group) across 23 studies	Preoperative imaging for ALT flap planning in reconstructive surgery	Anterolateral thigh (ALT) perforator flap using either color Doppler ultrasound (CDU) or computed tomography angiography (CTA) for perforator mapping	CDU had higher perforator detection sensitivity (95.3%) than CTA (90.4%); both had low false-positive rates (CDU: 2.8%, CTA: 2.4%); accuracy in course identification was high in both methods	Not directly discussed; focus was on diagnostic accuracy and imaging outcomes	Not applicable (review of preoperative imaging studies)	CDU offers slightly better sensitivity for ALT flap planning than CTA, though both imaging modalities are accurate and reliable; choice should be tailored to clinical needs and availability	10.1055/s-0041-1740958
4	36577497	Dow	2022	Outcomes of Free Muscle Flaps versus Free Fasciocutaneous Flaps for Lower Limb Reconstruction following Trauma: A Systematic Review and Meta-Analysis	Systematic review and meta-analysis	17 studies (retrospective studies, level three evidence, published between 1986 and 2021)	Lower limb reconstruction following trauma	Muscle free flaps (primarily latissimus dorsi) and fasciocutaneous free flaps (primarily anterolateral thigh (ALT))	No significant difference in total flap failure, reoperation, or limb salvage rates between muscle and fasciocutaneous flaps. Partial flap failure was lower in fasciocutaneous flaps.	No significant differences found in osteomyelitis, fracture union times, or functional recovery; partial flap failure rate was lower for fasciocutaneous flaps	Not specified	Both muscle and fasciocutaneous free flaps offer comparable outcomes for lower limb reconstruction following trauma, with fasciocutaneous flaps having lower partial flap failure rates	10.1055/a-2003-8789
5	31271453	Thomas	2020	Incidence of inadequate perforators and salvage options for the anterior lateral thigh free flap	Multicenter Study	1,079 cases of planned ALT flaps	Head and neck reconstruction	ALT free flap	28 ALT flaps discarded due to nonviable skin paddle or lack of perforators. Salvage options successfully utilized, with minimal additional morbidity.	No significant additional wound or morbidity in 12 salvage cases.	Not specified	ALT flaps are reliable, but when perforators are inadequate, multiple salvage options exist with minimal added morbidity if identified early.	10.1002/lary.28176

Study Number	PMID	First Author	Year	Title	Study Type	Sample Size	Indication	Flap Type / Technique	Main Outcomes	Complications	Follow-up Duration	Conclusion	DOI
6	31713920	Tsuge	2020	Preoperative vascular mapping for anterolateral thigh flap surgeries: A clinical trial of photoacoustic tomography imaging	Clinical Trial	8 patients (8 ALT flaps: 7 for head and neck reconstruction, 1 for chest wall reconstruction)	Head and neck reconstruction; chest wall reconstruction	Anterolateral thigh (ALT) flap; preoperative mapping using photoacoustic tomography (PAT)	Correlation between PAT-predicted and intraoperative perforator locations (within 10 mm); classification of perforator branching patterns	None of the 8 ALT flaps had postoperative complications	Not specified	PAT imaging accurately predicted perforator location (within 10 mm) and enabled preoperative vascular mapping, improving precision for ALT flap surgery planning.	10.1002/micr.30531
7	32712944	Ritschl	2020	Comparison between Different Perforator Imaging Modalities for the Anterolateral Thigh Perforator Flap Transfer: A Prospective Study	prospective study	12 patients	Preoperative perforator mapping for anterolateral thigh perforator flap (ALTPF)	Hand-held Doppler (hhD), Color Duplex Ultrasonography (CDU), and Indocyanine Green Angiography (ICGA)	ICGA detected 100% of perforators with highest accuracy; hhD sensitivity: 67%, PPV: 62%; CDU sensitivity: 73%, PPV: 64%	Not reported (focus on imaging accuracy)	Not applicable (intraoperative comparison)	ICGA was the most accurate and reliable method for perforator detection in ALT flap planning, outperforming hhD and CDU in sensitivity and PPV.	10.1055/s-0040-1714425
8	36150693	Illg	2023	Thermography Supported Color Duplex Ultrasound Accelerates ALT Perforator Imaging	Randomized controlled trial	32 healthy participants	Preoperative imaging for anterolateral thigh (ALT) perforator flap planning	Dynamic infrared thermography combined with duplex ultrasound	Thermography reduced ultrasound exam time by 90–130 seconds, Positive correlation between hotspot/perforator quantity and exam time	Not applicable (healthy volunteers, diagnostic study)	not applicable	Thermography enhances ALT perforator imaging by speeding up ultrasound exams and providing angiosome mapping insights.	10.1055/s-0042-1755614
9	29401129	Orseck	2018	Early Ambulation After Microsurgical Reconstruction of the Lower Extremity	Prospective analysis	36 free flaps (ALT and radial forearm)	Lower extremity reconstruction due to acute/chronic wounds and oncologic resection	Postoperative monitoring using physical exam, ViOptix (tissue oxygen saturation), and implantable venous Doppler	23 patients ambulated, 3 began leg dangling on POD1; one flap showed reversible early mottling; all flaps healed successfully	One reversible episode of flap mottling; no flap losses	Not explicitly stated	Multimodal flap monitoring can safely support early postoperative dependency protocols and improve detection of flap compromise early on.	10.1097/SAP.0000000000001346
10	35490120	de Berker	2022	Outcomes of microsurgical free tissue transfer performed on international surgical collaborations in low-income and middle-income countries: A systematic review and meta-analysis	Systematic review and meta-analysis	290 flaps on 284 patients	Head and neck (53%), lower limb (7.9%) reconstruction after cancer resection and trauma	Free tissue transfer during international collaborations	Flap failure rate: 3.8%, overall complication rate: 38%, emergency return to theatre rate: 19%, flap salvage success: 52%	Higher complication and take-back rates compared to higher-income settings	Not specified	Free flaps in LMICs have comparable failure rates to high-income settings but higher complication and take-back rates. Should inform preoperative counseling and consent.	10.1016/j.bjps.2022.04.002

Study Number	PMID	First Author	Year	Title	Study Type	Sample Size	Indication	Flap Type / Technique	Main Outcomes	Complications	Follow-up Duration	Conclusion	DOI
11	25824197	Matsuda	2015	Free perivascular tissue flap transfer	Case Series	7 patients (4 head and neck cases, 3 lower limb cases)	Soft tissue defects in the head and neck region, and lower limb (defects ranging from 4.0 cm³ to 40.0 cm³)	Free vascularized perivascular tissue flap based on the descending branch of the lateral femoral circumflex artery	Successful reconstruction of soft tissue defects, adjustable flap volume, rich vascular supply, combination with bone and cartilage tissue	Not specified	Not specified	The flap provides a versatile, easily elevated option for soft tissue reconstruction, overcoming limitations of local flaps and composite grafts.	10.1016/j.bjps.2015.03.005
12	36150694	Nagel	2023	From Esthetic Medicine to Optimizing Reconstructive Outcome: A Feasibility Trial on Secondary Refinement of Fasciocutaneous Anterolateral Thigh Flaps with Cryolipolysis	single-center, retrospective, interventiona l comparative cohort study	22 total (10 cryolipolysis; 12 surgical contouring)	Bulky free adipocutaneous ALT flaps after distal extremity reconstruction	Cryolipolysis (-9°C, 60 min) vs. standard surgical flap contouring	Cryolipolysis reduced extremity circumference (mean −1.8 cm) and fat thickness (−7.7 mm) significantly, 90% patient satisfaction, Shorter hospital stay vs. surgery ($p < 0.01$), One minor complication (frostbite) resolved conservatively	One case of second-degree frostbite; no major complications; compared favorably to surgical group	12 weeks post-procedure	Cryolipolysis is a promising, safe, and less resource-intensive alternative to surgical debulking of ALT flaps. Encourages future randomized controlled trials to validate findings.	10.1055/s-0042-1755259
13	31911012	Su	2020	Comparison of donor site complications of supra- versus subfascially harvested anterolateral thigh perforator free flaps: A meta-analysis	Systematic review and meta-analysis	525 patients from 7 studies	Reconstruction of defects	Comparison of supra-fascial (SPF) vs subfascial (SBF) anterolateral thigh (ALT) flap harvest techniques	No difference in need for skin grafting or sensory recovery, SPF group had lower rates of poor healing and donor site dysfunction	Donor site complications (healing issues and dysfunction) were more common with subfascial dissection	Not specified	Supra-fascial ALT flap harvest is associated with improved donor site healing and function, with similar outcomes in grafting and sensory recovery compared to subfascial harvest	10.1016/j.jcms.2019.11.023
14	29520951	Turin	2018	Decreasing ALT donor site morbidity with the keystone flap	retrospective, multi-institutional review	6 patients	Soft tissue reconstruction using anterolateral thigh (ALT) flap in cases where primary closure of the donor site was not possible	Anterolateral thigh (ALT) flap with donor site closure using a Keystone flap	Successful donor site closure without skin grafting; no major donor or recipient site complications; preserved hip and knee range of motion	One minor complication: delayed wound healing; no major complications observed	Not specified	Keystone flap is a safe and effective method for closing ALT donor sites where primary closure is not feasible, helping to avoid skin graft morbidity.	10.1002/micr.30317

Study Number	PMID	First Author	Year	Title	Study Type	Sample Size	Indication	Flap Type / Technique	Main Outcomes	Complications	Follow-up Duration	Conclusion	DOI
15	27567945	Irthum	2017	Anterolateral thigh flap for distal lower leg reconstruction	Retrospective, bicentric (two-centre) study	41 patients	Soft tissue defects of the distal lower leg (foot/ankle region) — the defect location was mainly on the foot and ankle (61 %) and bone exposure was found in 82.9 % of cases. The indication was trauma in 58.5 % of cases.	Free anterolateral thigh flap (ALT flap) used for reconstruction of the distal lower leg	Flap success rate: 92.8 %. Functional results rated "very good" or "good" in 75.5 % of cases.<br>Aesthetic results rated "very good" or "good" in 63.6 % of cases.	3 flap failures (7.3 %) and 3 patients lost to follow-up (7.3 %) were excluded from the functional/aesthetic outcome analysis	Not specified	The ALT flap demonstrated reliable results—with functional outcomes at least similar to other flaps—while maintaining low donor-site morbidity. Its versatility allows adaptation to the majority of lower-leg soft tissue defects.	10.1016/j.anplas.2016.07.017
16	39793965	Xu	2025	Clinical outcomes of reconstruction on tissue defects with thin anterolateral thigh flap versus commonly used free soft flaps: A meta-analysis	meta-analysis	416 cases from 7 published studies	Soft-tissue defects requiring free flap soft-tissue reconstruction; the analysis compares tALTF with other free soft flaps in reconstructive surgery.	The intervention was the free thin anterolateral thigh flap (tALTF). The comparator group was "commonly used free soft flaps	No significant difference in flap failure between tALTF vs CUFSFs.<br>No significant difference in the need for secondary salvage surgery.<br>tALTF exhibited significant advantages compared to CUFSFs in terms of: skin grafting requirement (less frequent), donor-site morbidity, recipient-site morbidity, and patient satisfaction.	While individual complication rates are not fully detailed in the abstract, the meta-analysis reports equivalent flap failure and salvage surgery rates for tALTF vs CUFSFs; improved donor/recipient site morbidity in favour of tALTF.	Not specified	The authors conclude that the thin anterolateral thigh flap (tALTF) is clinically safe and offers superior outcomes in several domains compared to commonly used free soft flaps in reconstructive surgery. They note, however, that the included studies are retrospective and carry limited sample sizes, so further large-scale prospective studies are needed to validate these findings.	10.1016/j.jormas.2025.102225
17	40598466	Liu	2025	Comparison between mixed reality with artificial algorithms and ultrasound in localization of anterior thigh flap perforators: a prospective randomized controlled study	prospective randomized controlled study	80 patients randomly assigned in MR or CDU group	Preoperative perforator vessel localization for anterolateral thigh (ALT) perforator flap surgery.	Anterolateral thigh perforator flap Technique comparison:<br>MR group: Mixed Reality (MR) + CT-angiography derived 3D model + artificial algorithm for vessel alignment and visualization.<br>CDU group: Standard preoperative Color Doppler ultrasonography (CDU) for perforator localization.	Primary outcomes:<br>Recognition (identification) rate of perforators: MR: 94.3%, CDU: 82.0%, P = 0.008<br>Accuracy of perforator location (distance between marked and actual exit points):<br>MR: 1.5 mm, CDU: 2.7 mm, P < 0.0001<br><br>Secondary outcomes:<br>Flap harvest time: MR: 52 min, CDU: 68 min, P < 0.0001<br>Flap survival: High survival in both groups with fewer complications in the MR group.	MR: 1 infection, 1 partial/total necrosis, CDU: 1 flap crisis, 2 flap necroses	Not specified	Integrating mixed reality (MR) with AI-based vessel matching algorithms significantly improves:<br>perforator recognition rate, localization accuracy, flap harvest efficiency, compared with standard CDU.<br>The technique shows strong potential for broader application in perforator-based reconstructive surgery across specialties.	10.1186/s12916-025-04181-0

Study Number	PMID	First Author	Year	Title	Study Type	Sample Size	Indication	Flap Type / Technique	Main Outcomes	Complications	Follow-up Duration	Conclusion	DOI
18	39542632	Han	2024	Effect of different degrees of wound eversion sutures on scar formation at donor site of anterolateral thigh flaps: A prospective randomized controlled study	prospective randomized clinical trial	Total: 36 patients, Completed follow-up: 30 patients Group A (non-eversion): n = 18, Group B (0.5cm eversion): n = 23, Group C (1.0cm eversion): n = 29	Donor site wound closure after ALT flap harvest; focus on scar formation	ALT flap, Wound closure at donor site with varying degrees of wound eversion: 0cm, 0.5cm, 1.0cm, Each patient's donor site incision was divided and randomly assigned to one of the eversion strategies	Scar assessment (6 months post-op): Patient and Observer Scar Assessment Scale (POSAS): Best outcomes in Group B (0.5 cm eversion), followed by Group A, worst in Group C, Patient satisfaction (VAS): Highest in Group B, Vancouver Scar Scale (VSS) & scar width: No significant differences among groups Healing: Most incisions healed by first intention. One poor healing and one delayed necrosis occurred in Group C, resolved with dressing/suturing.	Group C: 1 incision with poor healing, 1 incision necrosis at 3 months, resolved Groups A & B: No major complications; all other wounds healed primarily.	6 months postoperatively	Moderate wound eversion (0.5 cm) at the ALT donor site may reduce hypertrophic scar formation and improve patient satisfaction, compared to non-eversion or higher eversion (1.0 cm). Excessive eversion may not benefit scar outcomes.	10.7507/1002-1892.202406028

Supplementary Table S2: PRISMA checklist 2020

Section and Topic	Item #	Checklist item	Location where item is reported
**TITLE**			
Title	1	Identify the report as a systematic review.	1
**ABSTRACT**			
Abstract	2	See the PRISMA 2020 for Abstracts checklist.	1
**INTRODUCTION**			
Rationale	3	Describe the rationale for the review in the context of existing knowledge.	2
Objectives	4	Provide an explicit statement of the objective(s) or question(s) the review addresses.	2
**METHODS**			
Eligibility criteria	5	Specify the inclusion and exclusion criteria for the review and how studies were grouped for the syntheses.	3
Information sources	6	Specify all databases, registers, websites, organisations, reference lists and other sources searched or consulted to identify studies. Specify the date when each source was last searched or consulted.	3
Search strategy	7	Present the full search strategies for all databases, registers and websites, including any filters and limits used.	2-4
Selection process	8	Specify the methods used to decide whether a study met the inclusion criteria of the review, including how many reviewers screened each record and each report retrieved, whether they worked independently, and if applicable, details of automation tools used in the process.	2-4

Section and Topic	Item #	Checklist item	Location where item is reported
Data collection process	9	Specify the methods used to collect data from reports, including how many reviewers collected data from each report, whether they worked independently, any processes for obtaining or confirming data from study investigators, and if applicable, details of automation tools used in the process.	2-4
Data items	10a	List and define all outcomes for which data were sought. Specify whether all results that were compatible with each outcome domain in each study were sought (e.g. for all measures, time points, analyses), and if not, the methods used to decide which results to collect.	2-5
	10b	List and define all other variables for which data were sought (e.g. participant and intervention characteristics, funding sources). Describe any assumptions made about any missing or unclear information.	2-5
Study risk of bias assessment	11	Specify the methods used to assess risk of bias in the included studies, including details of the tool(s) used, how many reviewers assessed each study and whether they worked independently, and if applicable, details of automation tools used in the process.	2,3
Effect measures	12	Specify for each outcome the effect measure(s) (e.g. risk ratio, mean difference) used in the synthesis or presentation of results.	5
Synthesis methods	13a	Describe the processes used to decide which studies were eligible for each synthesis (e.g. tabulating the study intervention characteristics and comparing against the planned groups for each synthesis (item #5)).	2-5
	13b	Describe any methods required to prepare the data for presentation or synthesis, such as handling of missing summary statistics, or data conversions.	4-5
	13c	Describe any methods used to tabulate or visually display results of individual studies and syntheses.	5
	13d	Describe any methods used to synthesize results and provide a rationale for the choice(s). If meta-analysis was performed, describe the model(s), method(s) to identify the presence and extent of statistical heterogeneity, and software package(s) used.	3-5

**Section and Topic**	**Item #**	**Checklist item**	**Location where item is reported**
	13e	Describe any methods used to explore possible causes of heterogeneity among study results (e.g. subgroup analysis, meta-regression).	3-5
	13f	Describe any sensitivity analyses conducted to assess robustness of the synthesized results.	5
Reporting bias assessment	14	Describe any methods used to assess risk of bias due to missing results in a synthesis (arising from reporting biases).	5
Certainty assessment	15	Describe any methods used to assess certainty (or confidence) in the body of evidence for an outcome.	5
**RESULTS**			
Study selection	16a	Describe the results of the search and selection process, from the number of records identified in the search to the number of studies included in the review, ideally using a flow diagram.	3
	16b	Cite studies that might appear to meet the inclusion criteria, but which were excluded, and explain why they were excluded.	5-10
Study characteristics	17	Cite each included study and present its characteristics.	5-10
Risk of bias in studies	18	Present assessments of risk of bias for each included study.	9, Page 11-12 in Supplementary Files
Results of individual studies	19	For all outcomes, present, for each study: (a) summary statistics for each group (where appropriate) and (b) an effect estimate and its precision (e.g. confidence/credible interval), ideally using structured tables or plots.	5-10

Section and Topic	Item #	Checklist item	Location where item is reported
Results of syntheses	20a	For each synthesis, briefly summarise the characteristics and risk of bias among contributing studies.	4-9
	20b	Present results of all statistical syntheses conducted. If meta-analysis was done, present for each the summary estimate and its precision (e.g. confidence/credible interval) and measures of statistical heterogeneity. If comparing groups, describe the direction of the effect.	4-9
	20c	Present results of all investigations of possible causes of heterogeneity among study results.	9
	20d	Present results of all sensitivity analyses conducted to assess the robustness of the synthesized results.	9
Reporting biases	21	Present assessments of risk of bias due to missing results (arising from reporting biases) for each synthesis assessed.	11-12, Page 11-12 in Supplementary Files
Certainty of evidence	22	Present assessments of certainty (or confidence) in the body of evidence for each outcome assessed.	5-15
**DISCUSSION**			
Discussion	23a	Provide a general interpretation of the results in the context of other evidence.	16
	23b	Discuss any limitations of the evidence included in the review.	17
	23c	Discuss any limitations of the review processes used.	17
	23d	Discuss implications of the results for practice, policy, and future research.	17
**OTHER INFORMATION**			

Section and Topic	Item #	Checklist item	Location where item is reported
Registration and protocol	24a	Provide registration information for the review, including register name and registration number, or state that the review was not registered.	Was not registered
	24b	Indicate where the review protocol can be accessed, or state that a protocol was not prepared.	18
	24c	Describe and explain any amendments to information provided at registration or in the protocol.	18
Support	25	Describe sources of financial or non-financial support for the review, and the role of the funders or sponsors in the review.	18
Competing interests	26	Declare any competing interests of review authors.	18
Availability of data, code and other materials	27	Report which of the following are publicly available and where they can be found: template data collection forms; data extracted from included studies; data used for all analyses; analytic code; any other materials used in the review.	18

Supplementary Table S3: Risk of bias assessment:

Study (DOI)	Study Design	JBI Tool	Key Bias Issues	Overall Risk
10.1055/s-0040-1714425	RCT	JBI RCT Checklist	Unclear allocation concealment, no blinding	Moderate
10.1055/s-0042-1755614	RCT	JBI RCT Checklist	No blinding, unclear concealment, protocol reporting	Moderate
10.7507/1002-1892.202406028	RCT	JBI RCT Checklist	Attrition, unclear registration	Moderate
10.1186/s12916-025-04181-0	RCT	JBI RCT Checklist	Missing blinding, protocol transparency issues	Moderate
10.1002/micr.22492	Clinical Trial	JBI Quasi-Experimental	No randomization, confounding	Moderate
10.1002/micr.30531	Clinical Trial	JBI Quasi-Experimental	No control group, small sample	Moderate
10.1016/j.bjps.2015.03.005	Clinical Trial	JBI Quasi-Experimental	No control, confounding	Moderate–High
10.1097/SAP.0000000000001346	Clinical Trial	JBI Quasi-Experimental	No randomization, selection bias	Moderate–High
10.1055/s-0042-1755259	Clinical Trial	JBI Quasi-Experimental	Small feasibility study, no control	Moderate–High
10.1002/micr.30317	Clinical Trial	JBI Quasi-Experimental	Selection bias, no randomization	Moderate
10.1016/j.bjps.2017.04.001	Meta-analysis	JBI SR Checklist	Heterogeneity, publication bias	Moderate
10.1016/j.bjps.2022.04.002	Meta-analysis	JBI SR Checklist	Heterogeneous studies, limited bias assessment	Moderate
10.1016/j.jcms.2019.11.023	Observational	JBI Quasi-Experimental	Residual confounding	Low–Moderate
10.1002/micr.28176	Observational	JBI Quasi-Experimental	Selection bias, no randomization	Moderate
10.1016/j.anplas.2016.07.017	Clinical Trial	JBI Quasi-Experimental	No randomization, confounding	Moderate

Study (DOI)	Study Design	JBI Tool	Key Bias Issues	Overall Risk
10.1002/lary.28176	Clinical Trial	JBI Quasi-Experimental	No randomization, confounding	Moderate
